# Long-term patterns in European brown hare population dynamics in Denmark: effects of agriculture, predation and climate

**DOI:** 10.1186/1472-6785-4-15

**Published:** 2004-10-12

**Authors:** Niels M Schmidt, Tommy Asferg, Mads C Forchhammer

**Affiliations:** 1 Department of Ecology, Zoology Section, Royal Veterinary and Agricultural University, Thorvaldsensvej 40, DK-1871 Frederiksberg, Denmark; 2 Department of Population Biology, Institute of Biology, University of Copenhagen, Universitetsparken 15, DK-2100 Copenhagen, Denmark; 3 Department of Wildlife Ecology and Biodiversity, National Environmental Research Institute, Grenåvej 14, DK-8410 Rønde, Denmark

## Abstract

**Background:**

In Denmark and many other European countries, harvest records suggest a marked decline in European brown hare numbers, a decline often attributed to the agricultural practice. In the present study, we analyse the association between agricultural land-use, predator abundance and winter severity on the number of European brown hares harvested in Denmark in the years 1955 through 2000.

**Results:**

Winter cereals had a significant negative association with European brown hare numbers. In contrast to this, root crop area was positively related to their numbers. Remaining crop categories were not significantly associated with the European brown hare numbers, though grass out of rotation tended to be positively related. The areas of root crop production and of grass out of rotation have been reduced by approximately 80% and 50%, respectively, while the area of winter cereals has increased markedly (>70%). However, European brown hare numbers were primarily negatively associated with the number of red fox. Finally, we also found a positive association between mild winters and European brown hare numbers.

**Conclusion:**

The decline of Danish European brown hare populations can mainly be attributed to predation by red fox, but the development in agricultural land-use during the last 45 years have also affected the European brown hare numbers negatively. Additionally, though mild winters were beneficial to European brown hares, the increasing frequency of mild winters during the study period was insufficient to reverse the negative population trend.

## Background

In most countries in Western Europe, the landscape has undergone dramatic changes during the last century due to changes in the agricultural practices. In Denmark, both the uncultivated land and the semi-cultivated land, such as permanent grass areas, have been reduced dramatically, reflecting the general intensification of agriculture [1]. Additionally, fields have become larger, which has resulted in widespread fragmentation of remaining habitats, and today the landscape appears as a mosaic of natural habitats surrounded by cultivated land [1,2]. These changes in agriculture have affected a number of wildlife species living in this man-made landscape. For instance, the shift in agricultural practice has severely influenced the diversity and abundance of insects with concomitant consequences for the dynamics of a wide range of farmland birds [3,4], including the grey partridge (*Perdix perdix*) [5]. Among mammals, the European brown hare (*Lepus europaeus*) in particular has experienced a dramatic decline in many European countries [reviewed by [6]], including Denmark [7,8].

Despite its currently declining numbers, the European brown hare is still common and one of the most important game species throughout the country [8]. The dynamics of European brown hares seem resilient to even heavy hunting pressure [9], though local population dynamic data may be needed to ensure sustainable harvest [10]. In Denmark hunting of European brown hares is generally assumed to be without regulating effect [8]. The European brown hare is a typical grass steppe herbivore, and inhabits primarily open landscapes, including cultivated farmland [11], which is the predominant landform in Denmark [1]. The species is rather sedentary, and has generally small home ranges [e.g. [12]]. This site fidelity makes European brown hares highly susceptible to changes in their surrounding habitats, and the general decline in the European brown hare populations in Europe is mainly being attributed to changes in agriculture practice and land-use [reviewed by [6,12]].

European brown hares are important prey primarily for mammalian predators. In Northern Europe, the red fox (*Vulpes vulpes*) is the main predator on European brown hares, and foxes have been reported to influence the dynamics of several European brown hare populations substantially [13-17]. Also, infectious diseases such as the European brown hare syndrome virus, pseudotuberculosis, pasteurellosis and coccidiosis are present in many European brown hare populations [18-20]. Haerer et al. [19], however, concluded that diseases were not responsible for the decline of brown hare populations in Switzerland. Similarly, Frölich et al. [20] found that compared to red foxes, infectious diseases seemed to play a minor role in the dynamics of European brown hare populations in Germany.

An increasing number of papers have documented the importance of climate for a number of life history traits and abundance of many terrestrial vertebrates [21], and European brown hare populations are affected negatively by cold winters and cold springs [12,22-24]. Factors regulating vertebrate populations may, however, exhibit large spatial variation, and even among proximate populations spatial variation and gradients in vertebrate population dynamics may exist [e.g. [25]].

In this study, we analyse and contrast the simultaneous associations between agricultural land-use, the number of red fox harvested, winter severity and the population dynamics of European brown hares across 11 Danish districts during 45 years.

## Results

**Figure 1:**
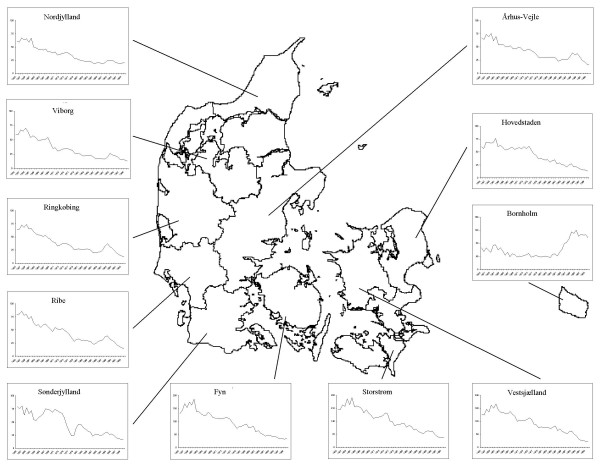
Harvest records of European brown hare (bags per 1000 ha) from 11 Danish districts, 1955–2000.

In the period 1955–2000 the European brown hare harvest record declined steadily in all the Danish districts but one: On the island Bornholm the European brown hare population declined until the late 1980s, but has increased markedly since then, and has now reached a level higher than that of 1955 (Fig. 1).

We found statistical significant direct density-dependence (*X*_*t-1*_) in the European brown hare time series (G = 281.4, df = 2, P < 0.0001). Additionally, the effect of district was also significant (G = 769.0, df = 1, P < 0.0001).

**Figure 2:**
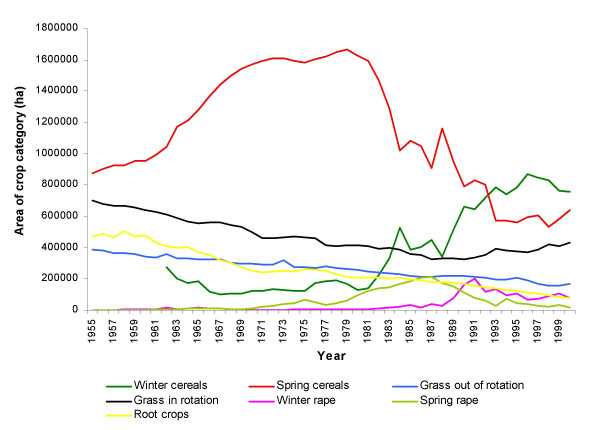
The total areas of the seven crop categories in Denmark 1955–2000. Note that prior to 1960 no data on winter cereals exist.

From 1955 to 2000, agricultural land-use changed markedly in Denmark, resulting in large temporal shifts in the areas covered by the different crop categories (Fig. 2). The areas covered with grass and green fodder in rotation and in particular the areas out of rotation have been reduced, the latter by approximately 50% since the mid 1950s. An even more dramatic decline has been observed for the root crops in the same period, a decline by more than 80% (Fig. 2). In Storstrøm, however, the area covered with root crops has been relatively stable over the years. In general, cereals were the dominant crop category in 1955 through 2000, with a shift from a predominance of spring cereals to winter cereals in the 1980s (Fig. 2).

The areas covered with winter cereals had a marked negative association with the number of European brown hares (Table 2), whereas root crops had a marked positive relation. Neither spring cereals, nor winter and spring rape seemed to be associated with European brown hare numbers (Table 2). Similarly, neither grass areas in or out of rotation were significantly related to European brown hare numbers, though the latter tended to have a positive association (Table 2).

**Table 2:** Summarised results from the analysis of the impact of agricultural land-use, red fox and winter climate on European brown hare harvest records from 11 Danish counties, 1955–2000. Also given is the change in model deviance (Δ deviance) explained by the variable when fitted last. Type 3 sums of squares.

Variable	Coefficient	SE	F value	P value	Δdeviance
Winter cereals_*(t)*_	-0.08322	0.03778	4.85	0.0282	37.7
Spring cereals_*(t)*_	-0.06243	0.04675	1.78	0.1825	2.5
Grass in rotation_*(t)*_	0.03217	0.03562	0.82	0.3669	4.1
Grass out of rotation_*(t)*_	0.05766	0.03070	3.53	0.0612	1.7
Winter rape_*(t)*_	-0.03272	0.03094	1.12	0.2909	4.0
Spring rape_*(t)*_	-0.02704	0.03948	0.47	0.4938	4.2
Root crops_*(t)*_	0.10370	0.04753	4.76	0.0297	0.4
Fox_*(t)*_	-0.01791	0.02024	0.78	0.3767	5.2
Fox_*(t-1)*_	-0.15720	0.01971	63.62	<.0001	52.8
NAO_*(t)*_	0.03732	0.01110	11.31	0.0008	4.0

Note: Due to the standardization of model variables, regression coefficients and Δ deviance values are directly comparable.

The number of red foxes harvested in year_*t-1 *_had a marked negative effect on the number of European brown hares harvested the following year (Table 2), whereas red fox number in year_*t *_seemed unimportant for the European brown hare numbers.

Mild winters, i.e. high winter state of the large-scale climatic phenomenon the North Atlantic Oscillation [NAO; [26]], had a significant positive effect on the European brown hare numbers (Table 2).

## Discussion

Despite its high reproductive potential [e.g. [27]], the Danish European brown hare has, according to annual harvest records, declined dramatically since 1955. Studies of Danish European brown hare populations indicate that its reproductive success is low compared to that of con-specifics in other countries [27], and has, in turn, declined from the 1950s to the 1990s [28]. Hansen [27] suggested that the low reproductive success observed in Danish European brown hares might be attributed to the agricultural practice. Using data covering almost half a century, our analyses suggest that the dramatic decline in the Danish European brown hares can be attributed mainly to the negative effect of red foxes, but also to the agricultural land-use. The area of winter cereal production has increased dramatically during the last century, and became the dominating crop in the early 1990s. We also found a significant positive association between root crops, a crop type that has declined dramatically in the second half of the last century, and European brown hare numbers.

European brown hares mainly forage on grasses and herbs [12], and cereals such as wheat are preferred during winter [29,30], which seems to contradict the results of our analyses. However, as European brown hares choose to feed on specific crops according to plant phenology [12,31], cereals, although important in winter, still occupy large areas when no longer of nutritional value to European brown hares, which may result in low availability of food especially during summer. Similarly, rape is avoided in the diet [29], but European brown hares may spend a substantial fraction of their time in rape fields during winter [30] prior to the development of glucosinulates [see [29]]. Apart from winter cereals and root crops, the crop categories did not affect the European brown hare numbers significantly. The lacking effect of grass and green fodder areas, especially those out of rotation, was unexpected as other studies have shown that hares prefer grass areas year-round [12,30]. This, however, may be attributed to the fact that we were unable to separate grass areas into those e.g. with and without cattle, factors that might affect European brown hare use of grass areas [12].

In a recent study, Fox [32] showed that farmland birds seemed to benefit from the reduced application of pesticides and inorganic fertilisers seen in Denmark since the early 1980s. The continuing decline in European brown hare numbers in that period therefore suggests that hares are not directly affected by the use of such substances, but rather respond to the loss of suitable habitat and space. European brown hares mainly move along field margins [12,33], and the increasing field size [34] combined with the general loss of suitable habitats possibly force hares to aggregate in the remaining patches of non-agricultural and non-urbanized land. Here, density-dependence may perpetuate the negative population development as European brown hares aggregate in the few, remaining pockets of suitable habitat. In line with this, Frylestam [23] reported an inverse relationship between fertility and density in European brown hares, which he attributed to shortage of food at least in some parts of the year, which also has been suggested in a number of other studies [12,24,27]. Hence, agricultural land-use, especially the increasing use of winter cereals and the marked reduction in root crops, but probably also grass areas out of rotation, seem to have contributed to the European brown hare decline in Denmark. However, the single-most important parameter for the number of European brown hares was the number of red foxes. Hence, our results are consistent with other studies reporting that red foxes may affect European brown hare populations negatively through predation [13-17]. This relationship is also particular evident from the positive development in the European brown hare population on the island Bornholm following the outbreak of sarcoptic mange there [35]. To elaborate the fox-hare interaction further, we reran the analyses including all variables but the red fox variables, and added the delayed AR term (i.e. *X*_*t-2*_). In seven of the 11 districts the delayed density dependence was significant (P < 0.05), suggesting important inter-specific interactions [36,37]. The significant association with red fox numbers in year_*t-1 *_(and not year_*t*_) most likely reflects that compared to adults, juvenile European brown hares suffer more from predation [e.g. [19]]. Hence, high red fox numbers in year_*t *_result in low harvest of European brown hares in year_*t*_, which affects the reproductive potential of the populations, and, hence, the number of European brown hares the following year (year_*t+1*_). Also, the significant effect of district may point to differences in habitat quality, but also differences in the history of the sarcoptic mange, i.e. time since the eruption of the mange, among districts [see [35]].

Both European brown hare over-winter survival [24] and reproductive rate [23] increases with temperature, which may be attributed to improved forage availability following mild winters [see [38,39]]. Our analyses revealed similar results as mild winters affected the European brown hares positively. There may, however, also be a negative effect of mild winters, namely through transmission of diseases and parasites, which may be enhanced under mild climatic conditions [18]. Nevertheless, the overall effect of mild winters seemed positive. The upward trend in the NAO since the 1960s [40], however, was not sufficient to reverse the European brown hare decline, but may have decelerated it.

## Conclusion

Our analyses have provided important insight into the structure of the European brown hare dynamics in Denmark, and documented important patterns within the mechanisms regulating European brown hares. Hence, we have documented that the decline of European brown hares in Denmark mainly can be attributed to predation by red fox, but also to changes in agricultural land-use. Additionally, though mild winters were beneficial to European brown hares, the increasing frequency of mild winters during the study period was insufficient to reverse the negative population trend.

## Methods

### European brown hare and red fox density indices

As indices of European brown hare and red fox density, we used the harvest records from 14 Danish counties from 1955–2000. In 1970, the geographical borders of some of the counties were changed, which in two cases lead to substantial changes in area [7]. This, together with inconsistency in crop registration, forced us to lump together some counties, and we therefore present analyses of European brown hare harvest records from 11 districts (Fig. 1). European brown hare and red fox harvest records were expressed as the number of animals shot per hectare per year. Harvest records may seem rather crude indicators of density compared to e.g. spotlight surveys and line transects [see [41]]. Unfortunately, no alternative indices of European brown hare density in Denmark are available. Harvest records, however, may still be useful indicators of the long-term trends in European brown hare numbers [41].

Sarcoptic mange was first encountered in Denmark in the early 1980s, and is now present in seven of the 11 districts [35]. In one district (Bornholm; Fig. 1), the disease has practically eliminated the entire red fox population, and red fox hunting has been prohibited here since 1991. Consequently, we restrict our analyses of the Bornholm district to 1955 through 1990. In all other districts, red fox hunting is open from 1 September to 31 January, and for European brown hares from 1 October to 31 December. Neither red fox nor European brown hare hunting is quota-based.

### Agricultural land-use

Time series quantifying agricultural land-use data, i.e. annual crop areas in hectares, covering the period 1955–81 were obtained from Statistics Denmark. Data for the period 1982–2000 were obtained from the official website of Statistics Denmark . The annual crop data were grouped into seven categories (Table 1).

**Table 1:** Description of model variables used in the analyses of European brown hare harvest records from Denmark, 1955–2000.

Variable	Description
Winter cereals	Winter cereals, including winter wheat, winter barley, and winter rye
Spring cereals	Spring cereals, including spring wheat, spring barley, spring rye, and oat
Grass in rotation	Grass and green fodder in rotation, including grass, clover, maize, and Lucerne
Grass out of rotation	Grass areas permanently out of rotation
Winter rape	Winter rape
Spring rape	Spring rape
Root crops	Sugar beets for sugar production and fodder, mangolds, turnips, and carrots for feed
Fox	The number of red fox harvested
NAO	The winter state of the North Atlantic Oscillation

No data on the fraction of winter and spring cereals, respectively, were available for 1955–61, and these data points were omitted in the analyses. Similarly, prior to 1982 no data on the fraction of winter barley and spring barley were available. However up until 1968, winter barley was only sown on a few thousand hectares in Denmark, when it was forbidden due to its function as reservoir for mildew attacking spring barley [42]. The use of mildew resistant winter barley was permitted in 1982. Hence, from 1955–81 barley was regarded as spring barley. Finally, no data on the fraction of winter and spring rye are available from 1955–61 and 1979–98, but in Denmark spring rye is generally sown on very few hectares only [42], and we regarded rye as winter rye unless otherwise specified [see also [32]]. Oat is sown as spring crop only. No data on the areas covered with grass and green fodder in or out of rotation were available for 1994, and the data point was interpolated, i.e. 1994 equalled the average of 1993 and 1995.

### Climatic variability

As an index of winter severity, we used the winter state of the North Atlantic Oscillation [NAO; [26]]. The climatic phenomenon NAO is defined as the difference in sea-level pressure between Portugal and Iceland, affecting the temperature, precipitation and wind across the Northern Hemisphere [26,43,44]. The NAO, thus, integrates the effects of a number of abiotic factors, and therefore seems particularly useful when analysing the dynamics of larger, endothermic animals with relatively large buffer-capacity against climatic variability.

The relationship between the winter NAO (December_*t-1 *_through March_*t*_) and temperature, precipitation and wind is particularly strong in Northern Europe [45]. When NAO is in its high state, the winter climate in Northern Europe is warm, wet, and windy, while when in its low state winters are cold and dry [44]. The NAO winter index has shown an upward trend since the 1960s, which accounts for a substantial fraction of the general warming in the Northern Hemisphere [40]. The NAO index data are available from the Climate Analysis Section, National Center for Atmospheric Research, USA .

### Data analyses

To remove heteroscedasticity in the system, data were log_e_-transformed, i.e. *X*_*(t) *_= ln(*N*_*(t) *_+ 1), where *N*_*(t) *_is the number of European brown hares harvested in year_*(t)*_. To allow the direct comparison of regression coefficients, variables were standardized prior to the analyses (i.e., [*X*_*(t)*_-*µ*_(1955–2000)_]/*s*_(1955–2000)_). Regression coefficients therefore express the rate of change in standard deviation units of the independent variable per one standard deviation unit of the dependent variable [46]. To obtain stationarity in the time series [see [47] for details], data were detrended following [48] by including year as covariate in all models.

For each district, we tested for the presence of multi-collinearity among parameters prior to the analyses by means of condition indices and variance proportions [49], but multi-collinearity was not observed in any districts (condition index < 12.47; [see [49] for details]).

The European brown hare time series were tested for non-linearity in *X*_*(t) *_vs. *X*_*(t-1) *_by applying the likelihood ratio test [50]. In all districts, linearity in *X*_*(t) *_vs. *X*_*(t-1) *_was not rejected λ< 7.76, P > 0.05). We analysed the data using a first-order autoregressive (AR(1)) mixed model approach with district as fixed factor:

*X*_*(t)*_~(*µ*,**V**),

where



and



where LAND_USE represents the seven crop categories (see Table 1). Because of the inclusion of red fox numbers, we did not include a delayed AR term (i.e. *X*_*t-2*_), as inclusion of both delayed density dependence and predator abundance can be seen as redundant [37]. All analyses were performed in SAS 8.2, using the PROC MIXED procedure with restricted maximum-likelihood estimation of regression coefficients [49]. Model reduction was conducted using log-likelihood ratio tests [51].

## Authors' contributions

NMS designed the study, participated in data preparation, carried out the statistical analyses, and drafted the manuscript. TA participated in data preparation. MCF supported the data analyses and contributed to the writing. All authors read and approved the final manuscript.
